# Astroglia acquires a toxic neuroinflammatory role in response to the cerebrospinal fluid from amyotrophic lateral sclerosis patients

**DOI:** 10.1186/s12974-016-0698-0

**Published:** 2016-08-30

**Authors:** Pooja-Shree Mishra, Dinesh K. Dhull, A. Nalini, K. Vijayalakshmi, T. N. Sathyaprabha, Phalguni Anand Alladi, Trichur R. Raju

**Affiliations:** 1 Department of Neurophysiology, National Institute of Mental Health and Neurosciences (NIMHANS), Bangalore, 560029 India; 2 Present address: Centre de Recherche de l’Institut Universitaire en Santé Mentale de Québec (CRIUSMQ), Québec, QC G1J 2G3 Canada; 3 Present address: Institute of Pharmaceutical Sciences, UGC-Center of Advanced Study (UGC-CAS), Panjab University, Chandigarh, 160014 India; 4 Department of Neurology, National Institute of Mental Health and Neurosciences (NIMHANS), Bangalore, 560029 India

**Keywords:** Neuroinflammation, Astrocytes, Cytokines, ROS, COX-2, Trophic factors, ALS

## Abstract

**Background:**

Non-cell autonomous toxicity is one of the potential mechanisms implicated in the etiopathogenesis of amyotrophic lateral sclerosis (ALS). However, the exact role of glial cells in ALS pathology is yet to be fully understood. In a cellular model recapitulating the pathology of sporadic ALS, we have studied the inflammatory response of astroglia following exposure to the cerebrospinal fluid from ALS patients (ALS-CSF).

**Methods:**

Various inflammatory markers including pro-inflammatory and anti-inflammatory cytokines, COX-2, PGE-2, trophic factors, glutamate, nitric oxide (NO), and reactive oxygen species (ROS) were analyzed in the rat astroglial cultures exposed to ALS-CSF and compared with the disease control or normal controls. We used immunofluorescence, ELISA, and immunoblotting techniques to investigate the protein expression and real-time PCR to study the messenger RNA (mRNA) expression. Glutamate, NO, and ROS were estimated using appropriate biochemical assays. Further, the effect of conditioned medium from the astroglial cultures exposed to ALS-CSF on NSC-34 motor neuronal cell line was detected using the MTT assay. Statistical analysis was carried out using one-way ANOVA followed by Tukey’s post hoc test, or Student’s *t* test, as applicable.

**Results:**

Here, we report that the ALS-CSF enhanced the production and release of inflammatory cytokines IL-6 and TNF-α, as well as COX-2 and PGE-2. Concomitantly, anti-inflammatory cytokine IL-10 and the beneficial trophic factors, namely VEGF and GDNF, were down-regulated. We also found impaired regulation of glutamate, NO, and ROS in the astroglial cultures treated with ALS-CSF. The conditioned medium from the ALS-CSF exposed astroglial cultures induced degeneration in NSC-34 cells.

**Conclusions:**

Our study demonstrates that the astroglial cells contribute to the neuroinflammation-mediated neurodegeneration in the in vitro model of sporadic ALS.

**Electronic supplementary material:**

The online version of this article (doi:10.1186/s12974-016-0698-0) contains supplementary material, which is available to authorized users.

## Background

Etiopathogenesis of amyotrophic lateral sclerosis (ALS), the devastating and relentlessly progressing neurodegenerative disorder leading to muscular weakness, is poorly understood. Approximately 90 % of the total cases reported have unknown etiology and are categorized as sporadic ALS. However, the rest 10 % of the cases follow an autosomal dominant inheritance pattern (familial ALS/FALS), and only ~20 % of these may be mapped to the mutations in the SOD-1 gene, which forms the basis of the animal models of ALS [1]. Apart from the occurrence of neuronal death, reports from these mutant SOD-1 transgenic models, as well as the autopsy studies, have demonstrated the non-cell autonomous contribution of the astrocytes in ALS [2–4].

The activated astrocytes may adopt either a neuroprotective or a neurotoxic phenotype in a stimulus-dependent manner by a process termed as glial polarization; the end-results of which depend largely on the microenvironment experienced by the astrocytes [5, 6]. For instance, a neuroprotective role of astrocytes has been thoroughly discussed in various pathological conditions including stroke and spinal cord injury [7]. On the other hand, astrocytes also respond in a toxic manner in response to excess ATP or the inflammatory factors like interleukin (IL)-1β and free radicals released by M1 microglia [8]. Apart from the increased expression of GFAP and S100β, activated astrocytes may respond by regulating certain inflammatory, trophic, and/or toxic factors in the milieu that may act directly on neurons or through other immune cells. These include pro/anti-inflammatory cytokines, inflammatory markers, and trophic factors [9, 10].

The plausible role of astrocytes in the initiation/progression of ALS has been studied employing chimeric mSOD1/TDP-43 models or human iPSC-derived astrocytes from ALS patients [11–13]. Such models have elucidated the possible toxic role of astrocytes in the pathophysiology of ALS. Lepore et al. [14] investigated the effect of transplantation of astroglial precursor cells into the spinal cord of mSOD-1 mice to establish healthy astroglial pools and demonstrated the mitigation of the disease symptoms, including a reduction in microgliosis. These findings suggest the important role of astrocytes in modulating the inflammatory response.

Glutamate-associated excitotoxicity following the selective loss of astroglial glutamate transporters, leading to reduced synaptic glutamate uptake, has been reported in the autopsy samples as well as the animal models of ALS [3, 15]. However, along with the reduction in glutamate uptake, possibility of pathological release of glutamate by astrocytes as the source of the abnormal elevation in the glutamate levels in ALS should also be considered. Some of the early work done indeed suggests excessive glutamate release in experimental models of familial ALS, but the source remains unknown [16, 17]. Additionally, glial cells including astrocytes are significant contributors to the oxidative stress, another proposed mechanism for the neurodegeneration in ALS [18]. However, the precise role of astroglia in ALS pathophysiology remains to be investigated. Further, most of the current understanding of the role of glia in ALS is derived from the animal models designed with genetic mutations/overexpression to recapitulate the disease symptoms of the familial form, thus precluding their relevance to the SALS pathogenesis.

To address this, we have established and characterized a cellular/animal model for SALS, which involves studying the toxic effect of the cerebrospinal fluid from the sporadic ALS patients (ALS-CSF). Such a model recapitulates the SALS pathology effectively. Using behavioral, immunocytochemical, molecular, electron microscopy and electrophysiological approaches, we have earlier demonstrated the potential of ALS-CSF to induce morphological and functional changes in motor neurons and glia. This was seen in neurons in the form of necroptosis-mediated neuronal death, reduced ChAT, Nav1.6 and Kv1.6 expression, aberrant phosphorylation of neurofilaments; mitochondrial, Golgi apparatus, ER and lysosomal defects, and reduced expression of trophic factors [19–29]. Concomitantly, infusion of ALS-CSF into the motor cortex of the adult rats caused altered electrophysiological properties of the motor neurons, as well as poor motor performance as seen with the rotarod and grip strength tests [30, 31].

Mass spectrometric analysis of the ALS-CSF demonstrated a clear up-regulation of glial inflammatory proteins including chitotriosidase, osteopontin, and chitinases 3 like protein-1 and 2 in the CNS circulation of the ALS patients, thus emphasizing the role of glia in SALS pathology [32]. We conducted the present study to determine the response of astrocytes following exposure to ALS-CSF, thereby elucidating their possible role in disease initiation and/or progression. To understand this, we have investigated the expression patterns of various cytokines, namely IL-6, tissue necrosis factor (TNF)-α, interferon gamma (IFN-γ), and IL-10, and trophic factors like vascular endothelial growth factor (VEGF) and glial cell line-derived neurotrophic factor (GDNF), as well as inflammation and toxicity mediators like prostaglandin E2 (PGE-2), cyclo-oxygenase 2 (COX-2), reactive oxygen species (ROS), nitric oxide (NO), and glutamate. Additionally, since our model largely takes into consideration the toxicity mediated by the circulating fluid of the CNS (ALS-CSF), we also investigated the effect of the conditioned media containing molecules secreted by astroglia, on cultured NSC-34 motor neuron cell line.

## Methods

### Cell culture

#### Primary astroglial cultures

Enriched astrocyte cultures were obtained from Wistar rats (P0-P2) using a modified protocol of Kerstetter and Miller [33]. Briefly, the spinal cords were dissected, freed of meninges, mechanically triturated in Dulbecco’s modified Eagle’s medium (DMEM), and propagated in DMEM with 10 % FBS (GIBCO-BRL). The mixed glial cultures thus obtained were allowed to attain 100 % confluence. On the 10th day in vitro (DIV), the cultures were spun at 200 rpm for 3–4 h in an incubated orbital shaker. The medium containing non-astroglial cells was discarded, and the enriched astroglial monolayer was trypsinized and replated at a density of 2.5 × 10^4^ cells/ml to avoid contamination with the non-astroglial cells, including microglia [34]. The astroglial cultures thus obtained were found to be >99 % pure (Additional file 1: Figure S1). Upon reaching 70–80 % confluence, the cultures were subjected to different experimental conditions.

#### NSC-34 cultures

The NSC-34 motor neuronal cell line was procured (Cedarlane, Canada), maintained, and propagated as per the published protocol [26]. For experiments, the NSC-34 cells were plated at a density of 2.5 × 10^4^ cells/ml in sterile 24-well plates and allowed to differentiate for 3–4 days in vitro, or until the cultures attained 70–80 % confluence. The cultures were then subjected to different experimental conditions as mentioned below.

### CSF collection and exposure

Six drug-naive patients diagnosed to have definite ALS and the age-matched neurological patients serving as disease control were selected for CSF collection. The samples were collected after obtaining informed consent as per the institutional human ethics committee guidelines. The diagnosis of ALS was based on the revised El Escorial criteria [35]. The mean age of the SALS patients was 47.17 years (Table 1). Age-matched NALS controls consisted of patients suffering from non-neurodegenerative/non-inflammatory neurological disorders including acquired peripheral neuropathy, idiopathic intracranial hypertension, and normal pressure hydrocephalus.

**Table 1:** Details of the ALS-CSF samples

Gender	Male, 4 (66.6 %)
	Female, 2 (33.3 %)
Diagnosis	Definite, 3 (50 %)
	Progressive, 3 (50 %)
Age at presentation (Mean ± SD)	47.17 ± 10.17 (38–65) years
Age at onset (Mean ± SD)	46.37 ± 9.98 (37.5–64) years
Duration of illness (Mean ± SD)	11.3 ± 6.6 (6.0–24) months
Onset pattern	Bulbar, 4 (66.6 %)
	Limb, 2 (33.3 %)

The samples were snap frozen in liquid nitrogen and stored at −80 °C until further use. For the experiments, the cultures were exposed to 10 % *V*/*V* CSF in DMEM, and the effects were studied in duplicates for all the six CSF samples discretely. Thus, depending on the experiments, a minimum of three and a maximum of six separate cultures were studied in replicates for each experimental group. The study consisted of three experimental groups, namely:

1. Normal control (NC): cultures propagated in DMEM
2. NALS (disease control): cultures exposed to CSF from the age-matched patients suffering from neurological diseases such as intracranial hypertension (NALS-CSF, 10 % *V*/*V* in DMEM) other than neurodegenerative and neuroinflammatory disorders
3. ALS: cultures exposed to CSF from ALS patients (ALS-CSF; 10 % *V*/*V* in DMEM)

### ELISA

ELISA was used to quantify IL-6, IL-10, IFN-γ, and TNF-α expression in the culture medium. Specific rat ELISA kits were procured from RayBiotech, Inc (Georgia, USA), and the standards were prepared according to the manufacturer’s instructions. For a temporal analysis, the cultures were exposed to CSF for 12, 24, and 48 h, respectively, and the media from the cultures was used for the estimation.

### Glutamate estimation

Approximately 10^6^ cells were harvested from the astroglial cultures exposed to ALS-CSF or the normal controls for 48 h, and lysates were prepared. Simultaneously, the medium from the normal controls and each of the subsets exposed to ALS-CSF for 12, 24, and 48 h was collected and centrifuged at 14,000 rpm to remove the debris. The cytosolic and secreted glutamate levels were measured using the glutamate assay kit (MAK004, Sigma-Aldrich, USA). The measurement was based on enzymatic conversion, and the absorbance was read at 450 nm using a colorimetric ELISA microplate plate reader (Tecan 2500 fluorometer, USA).

### Nitric oxide estimation

The lysates from the astroglial cultures exposed to ALS-CSF for 48 h or the normal controls were prepared as described above. Simultaneously, the medium from the normal cultures and those exposed to ALS-CSF was also collected. The level of nitrates was assayed in the samples using nitric oxide assay kit (AB65328, Abcam) at 540 nm using a colorimetric ELISA microplate reader (Tecan 2500 fluorometer, USA).

### ROS estimation

The ROS levels were measured in astroglial cultures exposed to ALS-CSF using dichlorofluorescin diacetate (DCFDA), a compound that reacts with the intracellular ROS to produce fluorescence. Briefly, the cultures were treated with DCFDA, harvested, and lysed, and the resultant fluorescence release was measured at 536 nm in an ELISA plate reader. Fluorescence/cell was calculated and statistically analyzed. For qualitative studies, the cells were plated onto 13-mm coverslips. After exposure, the cells were incubated with Locke’s solution and DCFDA and directly viewed (excitation—480 nm; emission—530 nm) using confocal microscopy (Leica TCS-SL, Leica Microsystems, Germany).

### MTT assay

Cell viability for NSC-34 cell line was assayed using MTT assay [36]. Briefly, after 48 h of exposure to the astroglial-conditioned media, NSC-34 cells were treated with MTT solution (5 mg/ml, 37 °C). After 2 h, dimethyl sulfoxide (DMSO) was added to solubilize the resultant MTT formazan. Absorbance was measured at 570 nm using an ELISA plate reader, and the percentage of viable cells was statistically analyzed (Tecan 2500 fluorometer, USA).

### Quantitative immunofluorescence

Cellular localization, as well as the expression of inflammatory molecules and trophic factors, was studied using quantitative immunofluorescence. Briefly, astroglial cultures were plated at a density of 2.5 × 10^4^ cells/ml onto the 13-mm circular coverslips pre-coated with poly-l-lysine (0.1 mg/ml). These were exposed to the aforementioned experimental conditions for 48 h, after which the cells were fixed with 4 % paraformaldehyde (PFA) for 15 min at RT. Blocking was carried out with 3 % bovine serum albumin (BSA) and incubated with primary antibodies of interest for 24 h, followed by appropriate secondary antibodies tagged with FITC or CY3 for 2 h at RT. The specificity was ensured for both primary and secondary antibodies by adding relevant positive and/or negative controls to the experiments. For double immunofluorescence, the coverslips were re-equilibrated with 0.1 M PBS (pH 7.4) and blocked with 3 % BSA, following which the second set of primary and secondary antibodies were added. The list of antibodies, the dilution factor, incubation time, and the temperature are provided in Table 2. Finally, the coverslips were mounted using PVA-DABCO (Sigma-Aldrich, USA) and viewed under the laser scanning confocal microscope (Leica TCS-SL, Germany), with excitation wavelengths at 488 and 514 nm for FITC and Cy3, respectively. The emission frequencies were segregated to avoid non-specific overlap of labeling. The images thus captured were quantified for the fluorescence intensities of each immuno-labeled protein using the inbuilt software of Leica Microsystems [25, 37]. Briefly, 8-b images were captured at ×20 magnifications with a constant PMT voltage, from randomly selected 10 non-overlapping fields in each coverslip. Other parameters like objective (20×), optical zoom of 2×, pinhole [airy] (1.000075), frame average (3), line average (3), resolution (8), frame (1024 × 1024), and exposure time were also kept constant. We measured the fluorescence intensity graded on a scale of 0–255, where “0” refers to minimum fluorescence and “255” refers to maximum fluorescence on an 8-b image. The region of interest (ROI), i.e., the region of the each cell where the staining was prominently seen, was drawn/demarcated using the “poly-line” profile of the inbuilt software by Leica. Each cell represented a single ROI, and a minimum of 20 such cells were quantified for each image. Thereafter, the image was subjected to analysis using the inbuilt software, which generated the numerical values commensurate to the staining. At least 10 such images for each coverslip were randomly analyzed, thus measuring intensities for least 200 cells per coverslip (Additional file 2: Figure S2). Each coverslip corresponded to one replicate of a sample. Five such samples were analyzed in duplicates for all the three experimental subsets. Background reduction was applied for each analysis, and the results were then compared.

**Table 2:** List of the antibodies used in the study

Primary antibody	Secondary antibody
Anti-COX-2 rabbit polyclonal (1:500, Abcam); 24 h, 4 °C	Anti-rabbit IgG (Cy3-conjugated; 1:200, Sigma-Aldrich) 2 h, RT
Anti-GDNF mouse monoclonal (1:200, SCBT), 24 h, 4 °C	Anti-mouse IgG (Cy3-conjugated; 1:200, Sigma-Aldrich) 2 h, RT
Anti-IFN-γ goat polyclonal (1:200, SCBT) 24 h, 4 °C	Anti-goat IgG (Cy3-conjugated; 1:200, Sigma-Aldrich) 2 h, RT
Anti-IL-10 rabbit polyclonal (1:500, Abcam) 24 h, 4 °C	Anti-rabbit IgG (Cy3-conjugated; 1:200, Sigma-Aldrich) 2 h, RT
Anti-IL-6 rabbit polyclonal (1:500, Abcam) 24 h, 4 °C	Anti-rabbit IgG (FITC-conjugated; 1:200, Chemicon) 2 h, RT
Anti-iNOS rabbit polyclonal (1:800, Abcam) 24 h, 4 °C	Anti-rabbit IgG (Cy3-conjugated; 1:200, Chemicon) 2 h, RT
Anti-PGE-2 rabbit polyclonal (1:500, Abcam) 24 h, 4 °C	Anti-rabbit IgG (FITC-conjugated; 1:200, Chemicon) 2 h, RT
Anti-TNF-α mouse monoclonal (1:200, Abcam), 24 h, 4 °C	Anti-mouse IgG (Cy3-conjugated; 1:200, Sigma-Aldrich) 2 h, RT
Anti-VEGF rabbit polyclonal (1:500 Abcam), 24 h, 4 °C	Anti-rabbit IgG (FITC-conjugated; 1:200, Chemicon) 2 h, RT

### Immunoblotting for COX-2, VEGF, and GDNF

Following 48 h of CSF exposure, the astroglial cells were lysed on ice using cell disruption buffer (AMBION, USA containing 0.1 % protease inhibitor cocktail (Sigma-Aldrich)). The lysates thus obtained were centrifuged at 12,000 rpm for 15 min at 4 °C. The suspension was carefully collected, and the total protein concentration was determined using Bradford’s reagent. Twenty micrograms of the protein sample was loaded for each sample. The proteins were separated on 10 % denaturing gel for COX2 and VEGF and 12 % denaturing gel for GDNF, in accordance with the predicted molecular weight. The proteins were then transferred to PVDF membrane. The membrane was then subjected to blocking with 7 % skimmed milk overnight at 4 °C. The blots were further incubated overnight at 4 °C with mouse monoclonal anti-GDNF (1:1000, SCBT), Rabbit polyclonal anti-VEGF (1:1000, Abcam), or Rabbit polyclonal anti-COX-2 (1:1000, Abcam) depending on the experiments, followed by specific biotinylated anti-mouse for anti-GDNF (1:5000, Vector Laboratories) and anti-rabbit antibody for anti-VEGF and anti-COX-2 (1:5000, Vector Laboratories) for 2 h. The blots were developed using Avidin-Biotin complex and developing solutions (ABC-AP kit, Vector laboratories). The bands were visualized, and the intensity was quantified using the Gel Documentation System (Quantity 1 software, Biorad, USA). β-Actin (Sigma-Aldrich) was used as the loading control, and the band intensity for the protein of interest was normalized to that of the β-actin protein.

### Quantitative RT-PCR

Upon exposure to CSF for 48 h, the astroglial cells were collected and the total RNA was isolated using RNeasy Plus Mini Kit (Qiagen, USA) as per the manufacturer’s instructions. The messenger RNA (mRNA) was quantified using 1 μl of RNA in a NanoDrop 2000 spectrophotometer (Thermo Fisher Scientific Inc). Approximately 10 ng of RNA was reverse transcribed (RT) using a high-capacity complementary DNA (cDNA) reverse transcription kit (Applied Biosystems, USA). Real-time PCR amplification was carried out in triplicates using specific primers and hydrolysis probes for the target genes listed in Table 3, as well as 18S (endogenous control) in a Rotor Gene-6000 real-time PCR cycler. The reaction efficiency for the target genes and 18S was assayed and compared using the standard curve method, and the assays were standardized until the PCR efficiency of ~100 ± 10 % was achieved. The cycle threshold (Ct) values were normalized to the endogenous control 18S. The relative fold change was calculated using the comparative CT method (ΔΔCT method) [38].

**Table 3:** Details of the primers and probes used in the study

Gene	Sense primer	Anti-sense primer	Probe
VEGF	GAGCAACGTCACTATCGAGATC	GGCTTTGTTCTATCTTTCTTGGTC	TGCCGATCAAACCTCACCAAAGCCA
GDNF	CGCCGGTAAGAGGCTTCTC	GATAATCTTCGGGCATATTGGAGTC	CGCCCGCCGAAGACCACTCCCT
IL-6	TCCAGCCAGTTGCCTTCTTG	TCCTCTGTGAAGTCTCCTCTCC	ACTGATGTTGTTGACAGCCACTGCCTTCC
IL-10	CATGGCCTTGTAGACACCTTTG	CATCGATTTCTCCCCTGTGAGA	TCATTCTTCACCTGCTCCACTGCCTTGCTT
IFN-γ	GCACAAAGCTGTCAATGAACTCA	CCAGAATCAGCACCGACTCC	CTGTCACCAGAATCTAGCCTAAGGAAGCGG
COX2	CCAACCTCTCCTACTACACCAG	GTTCCTTATTTCCTTTCACACCCATG	CCTTCCTCCTGTGGCTGATGACTGC
iNOS	CATCGACCTGGGCTGGAA	CCTCTGGATCTTGACCGTGAG	CGATGTGCTGCCTCTGGTCCTGC

### Statistical analysis

The data for mRNA expression, quantitative immunofluorescence, semi-quantitative western blots, and MTT assay was statistically assessed for significance by one-way ANOVA followed by Tukey’s post hoc test. For ELISA, glutamate, NO, and ROS, the statistical analysis was carried out using Student’s *t* test. GraphPad Prism was the software used to analyze the significance.

## Results

### ALS-CSF exposure impairs regulation of pro and anti-inflammatory cytokines

**Fig. 1:**
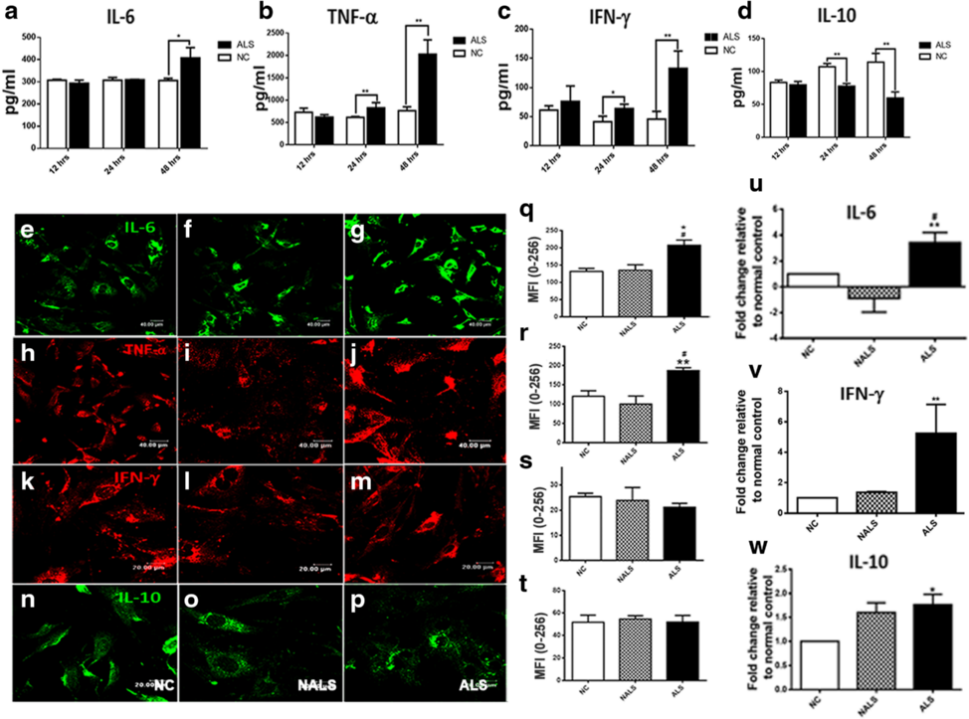
Pro-inflammatory cytokines are up-regulated in response to ALS-CSF: exposure of astrocytes to ALS-CSF caused a progressive up-regulation in the secretion of IL-6 (**a**; *p < 0.05 NC v/s ALS, 48 h; *n* = 3 in duplicates), TNF-α (**b**; **p < 0.01 NC v/s ALS, 24 and 48 h; *n* = 3 in duplicates), and IFN-γ (**c**; *p < 0.05, 24 h and **p < 0.01 NC v/s ALS, 48 h; *n* = 3 in duplicates), while a down-regulation in the IL-10 levels was observed (**d**; **p < 0.01 NC v/s ALS, 24 and 48 h; *n* = 3 in duplicates). The protein levels were determined by ELISA. **e**–**p** The confocal images representing the cellular expression of the cytokines in the normal controls as compared to the cultures exposed to NALS/ALS-CSF. Analysis of the mean fluorescence intensity (MFI) revealed a qualitative as well as a quantitative increase in the expression of IL-6 (*green*, **e**–**g**; graph **q**; *p < 0.05 NC v/s NALS and ALS; *n* = 5 in duplicates) and TNF-α (*red*, **h**–**j**; graph **r,** #*p* < 0.05 NC v/s ALS and **p < 0.01 NALS v/s ALS; *n* = 5 in duplicates). However, the cellular levels of IFN-γ (*red*, **k**–**m**; graph **s**; *n* = 5 in duplicates) and IL-10 (*green*, **n**–**p**; graph **t**; *n* = 5 in duplicates) did not show any significant changes in the cultures exposed to ALS-CSF. Graphs **u**–**w** represent the mRNA expression levels of IL-6, IFN-γ, and IL-10, respectively, in response to ALS-CSF. Note the up-regulation in IL-6 (**p < 0.01 NC and ^#^ *p* < 0.05 NALS v/s ALS; *n* = 3 in triplicates; **u**), IFN-γ (**p < 0.01 NC and NALS v/s ALS; *n* = 3 in triplicates; **v**), and IL-10 mRNA expression (*p < 0.05 NC v/s ALS; *n* = 3 in triplicates; **w**) when exposed to ALS-CSF. Analysis of significance was carried out using Student’s *t* test for ELISA and one-way ANOVA followed by Tukey’s post hoc test for determining the MFI as well as the mRNA expression. *Scale bars* for confocal images are indicated

The temporal effect of ALS-CSF on the release of cytokines was determined at three different time points viz., 12, 24, and 48 h. ALS-CSF exposure resulted in the enhanced release of pro-inflammatory cytokines IL-6, TNF-α, and IFN-γ from the astrocytes as compared to the normal controls (Fig. 1). Increased IL-6 secretion was observed at 48 h (*p < 0.05 NC v/s ALS; Fig.1a), whereas TNF-α (**p < 0.01 NC v/s ALS, 24 and 48 h; Fig. 1b) and IFN-γ (*p < 0.05 NC v/s ALS, 24 h and **p < 0.01 NC v/s ALS, 48 h; Fig. 1c) were up-regulated as early as 24 h with the levels peaking at 48 h. Concomitantly, a reduction in the levels of IL-10 was noted beginning at 24 h in cultures exposed to ALS-CSF as against the normal controls (**p < 0.01 NC v/s ALS, 24 and 48 h; Fig. 1d). A similar effect was also observed in the cellular levels of the cytokines. Quantification of the mean immunofluorescence intensity of cultures immunostained for IL-6 and TNF-α proteins (Fig. 1e–g, graph q and h–j, graph r, respectively), confirmed the increased expression of both these proteins in the ALS subset after 48 h as compared to the NC as well as the disease (NALS) controls (*p < 0.05 NC and ^##^*p* < 0.01 NALS v/s ALS). However, the cellular level of IFN-γ was stable (Fig. 1k–m, graph s). At 48 h, the cellular IL-10 also remained unaffected, although a minor down-regulation was noted (Fig. 1n–p, graph t).

Analysis of the mRNA expression of cytokines at 48 h revealed a sizeable up-regulation of IL-6 (Fig. 1u) and IFN-γ (Fig. 1v).In cultures exposed to ALS-CSF, we observed nearly a threefold up-regulation in IL-6 expression (**p < 0.01 NC and ^#^*p* < 0.05 NALS v/s ALS) and approximately fivefold in IFN-γ expression (**p < 0.01 NC and NALS v/s ALS) when compared to the NC subset. Surprisingly, IL-10 mRNA levels were up-regulated nearly 1.5fold, when compared to NC (*p < 0.05 NC v/s ALS; Fig. 1w).

### COX-2 and PGE-2 expression were up-regulated in cultures exposed to ALS-CSF

**Fig. 2:**
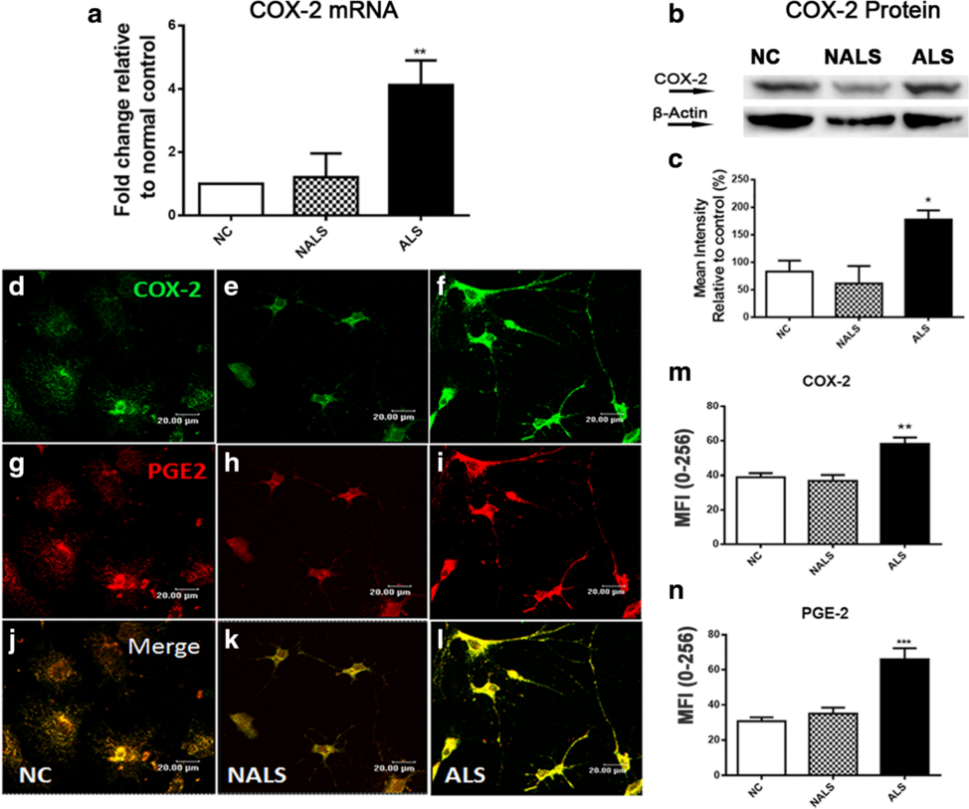
ALS-CSF induces inflammatory response by astrocytes: COX-2 mRNA expression was seen up-regulated in response to ALS-CSF (**p < 0.01 NC and NALS vs. ALS, *n* = 5 in triplicates; graph **a**), suggesting the induction of neuroinflammatory pathways. Note the significant up-regulation in the COX-2 protein expression in the ALS group, as observed by western blot analysis (**b**,*p < 0.05 NC and NALS vs. ALS; *n* = 3; graph **c**). Representative confocal images of the cultures immunostained for COX-2 (FITC, *green*; **d**–**f**; **p < 0.01 NC and NALS vs. ALS; *n* = 5 in duplicates; graph **m**) and its downstream inflammatory molecule PGE-2 (Cy3, *red*, **g**–**i**; ***p < 0.001 *n* = 5 in duplicates; graph **n**) further demonstrate the inflammatory potential of the astroglial cells in response to ALS-CSF. Analysis of the significance was carried out using one-way ANOVA followed by Tukey’s post hoc test. *Scale bars* are indicated

We also studied the expression pattern of both COX-2 and PGE-2 in astrocyte cultures following exposure to ALS-CSF. We observed a significant up-regulation in the mRNA expression of COX-2 (**p < 0.01 NC and NALS vs. ALS; Fig. 2a). The immunofluorescence pattern revealed that COX-2 and PGE-2 exhibited a punctate cytoplasmic staining in astrocytes (Fig. 2d–l). Quantification of the mean fluorescence intensity also showed a significant increase in the levels of COX-2 protein expression as compared to NC and NALS subsets (**p < 0.01 NC and NALS v/s ALS; Fig. 2m). The results were further validated by the increased expression of COX-2 in ALS-CSF compared to the normal and disease control, as determined by the western blot analysis (*p < 0.05 NC and NALS v/s ALS; Fig. 2b, c). In a similar manner, ALS-CSF caused up-regulation of PGE-2 levels, while NALS-CSF did not, and was comparable to the normal controls (***p < 0.001 NC and NALS v/s ALS; Fig. 2n).

### ALS-CSF enhances production and release of astroglial glutamate

**Fig. 3:**
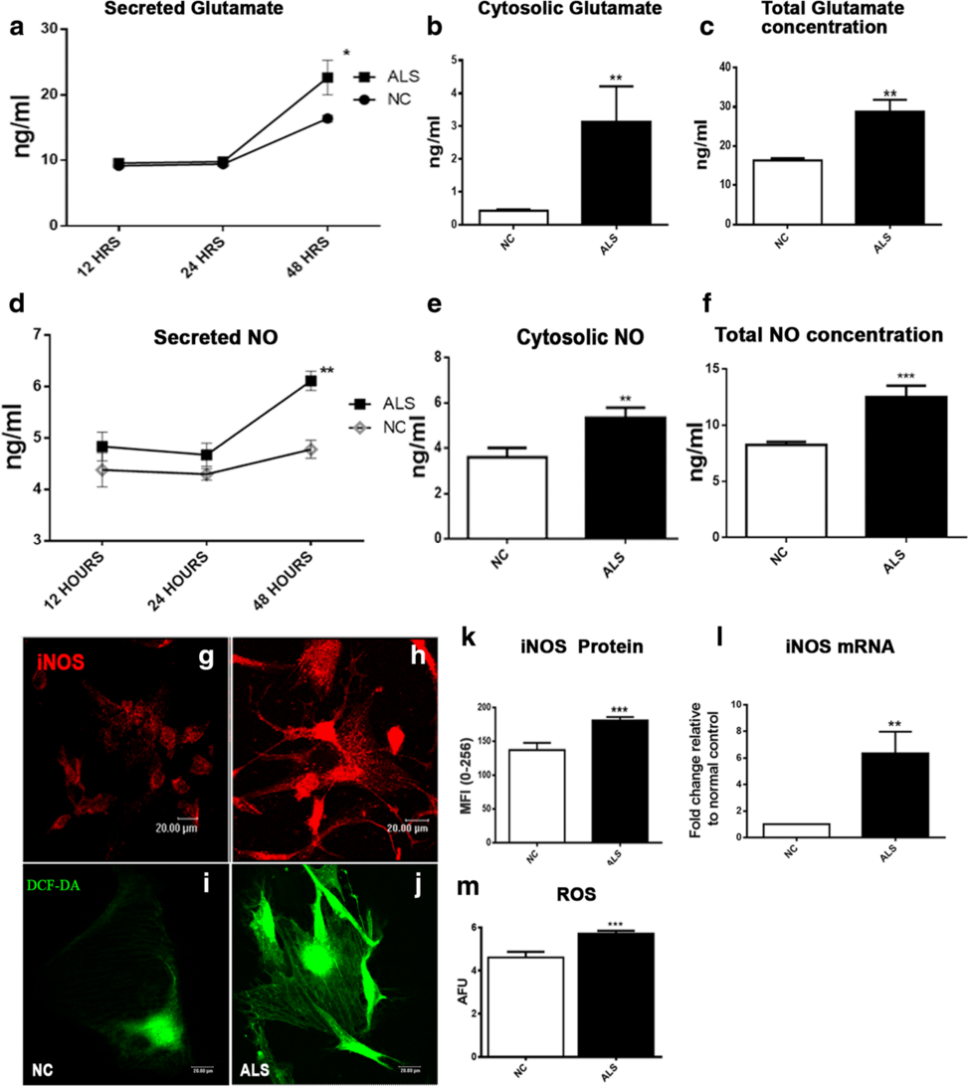
Astrocytes secrete increased levels of glutamate, ROS, and NO in response to ALS-CSF and impart neurotoxicity to NSC-34 cells. **a**–**c** Graphs representing the up-regulation in the glutamate synthesis and secretion by astrocytes. Glutamate secretion was up-regulated in the astroglial supernatants 48 h after the exposure to ALS-CSF as compared to NC (*p < 0.05 NC v/s ALS; *n* = 5 in duplicates; graph **a**). Also, note the up-regulated expression in the cellular glutamate (**p < 0.01 NC v/s ALS; *n* = 5 in duplicates; graph **b**). Taken together, the total increase in the astroglial glutamate levels is evident as plotted in graph **c** (**p < 0.01 NC v/s ALS; *n* = 5 in duplicates). Similarly, the secreted (**p < 0.01 NC v/s ALS 48 h, *n* = 5 in duplicates, graph **d**), cellular (**p < 0.01 NC v/s ALS, *n* = 5 in duplicates, graph **e**), and total expression levels (***p < 0.001 NC v/s ALS; *n* = 5 in duplicates; graph **f**) of NO showed up-regulation in response to a 48-h exposure to ALS-CSF as compared to NC. The confocal images **g**, **h** (CY3, *red*) represent the expression patterns of iNOS in the NC and ALS subsets, respectively. Note the enhanced expression of iNOS in ALS group, as determined by the MFI (***p < 0.001 NC v/s ALS; *n* = 5 in duplicates, graph **k**). Also note the increased mRNA expression of iNOS in response to the ALS-CSF as compared to the normal controls (**p < 0.01 NC v/s ALS; *n* = 3 in triplicates; graph **l**). Representative confocal images for the cellular levels of (DCFDA, *green*, **i**, **j**) as well as quantitative fluorometric analysis (graph **m**) denote an increased ROS expression in ALS subsets as compared to the NC (***p < 0.001 NC v/s ALS; *n* = 6 in triplicates). Analysis of significance was carried out using Student’s *t* test. *Scale bars* are indicated

We compared the levels of astroglial glutamate upon exposure to ALS-CSF (Fig. 3a–c). The secreted glutamate levels were significantly elevated in ALS subsets as compared to the normal controls only at 48 h (*p < 0.05 NC v/s ALS), but not at 12 or 24 h (Fig. 3a). The astroglial lysates too showed a significant increase in the glutamate levels when compared to the normal control (**p < 0.01 NC v/s ALS; Fig. 3b). Thus, a concomitant increase in the cytosolic, as well as secreted glutamate levels, was seen in the ALS group by 48 h (Fig. 3c and Table 4). This observation strongly suggests excessive glutamate production, in addition to secretion occurring in response to ALS-CSF.

**Table 4:** Summary of the astroglial cytosolic and secreted glutamate levels (ng/ml) at 48 h after the CSF exposure

	NC	ALS
Cytosolic	0.42 ± 0.04	3.13 ± 1.08**
Secreted	16.18 ± 0.53	24.25 ± 3.279*
Overall	16.62 ± 0.57	28.82 ± 3.04**

Values are expressed as Mean ± SEM

*p < 0.05; **p < 0.01

### ALS-CSF induced enhanced release of astroglial nitric oxide and ROS

We then analyzed the astroglial ROS and NO levels, considering the inflammatory and toxic phenotype adopted by the astrocytes in response to ALS-CSF. Analysis of lysates revealed a significant increase in the amount of cytosolic NO in astroglial cultures exposed to ALS-CSF at 48 h, when compared to the normal controls, but not at 12 or 24 h (**p < 0.01 NC v/s ALS; Fig. 3d, Table 5). The secreted NO levels were also higher in the ALS-CSF exposed cultures lysates at 48 h (**p < 0.01 NC v/s ALS; Fig. 3e and Table 5). To determine the source of the pathological amounts of NO, the expression of iNOS was studied. Analysis of the mRNA expression (**p < 0.01 NC v/s ALS; Fig. 3l) and quantitative analysis of the mean immunofluorescence intensity demonstrated an up-regulation of the iNOS protein in ALS subset compared to the normal controls (***p < 0.001 NC v/s ALS; Fig.3k).

**Table 5:** Summary of the cytosolic and secreted levels of NO (ng/ml), 48 h after the exposure to ALS-CSF

	NC	ALS
Cytosolic	3.59 ± 0.41	5.3 ± 0.44 **
Secreted	4.96 ± 0.17	6.11 ± 0.18**
Overall	8.22 ± 0.27	12.50 ± 1.0***

Values are expressed as Mean ± SEM

**p < 0.01; ***p < 0.001

Qualitative assessment of the cytosolic ROS by confocal microscopy also revealed enhanced ROS levels in cultures exposed to ALS-CSF for 48 h when compared to the normal control (Fig. 3i, j). Further, a quantitative fluorometric assessment of the ROS levels in NC and ALS cellular lysates showed a significantly higher ROS levels in the ALS group (***p < 0.001 NC v/s ALS; graph m).

### Decline in trophic factor expression accompanied neuroinflammation

**Fig. 4:**
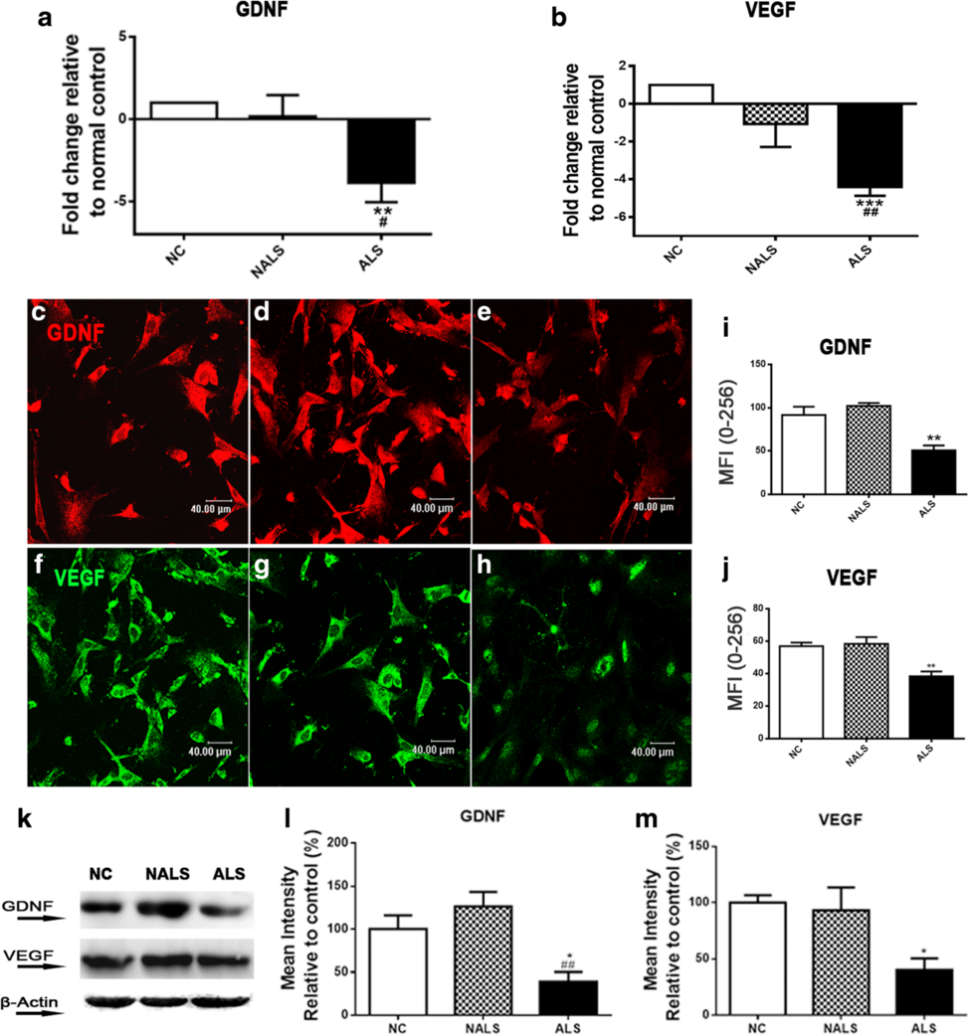
Down-regulated trophic factor expression in response to ALS-CSF. Graphs **a**, **b** denote the down-regulation in the mRNA levels of GDNF (**p < 0.01 NC and ^#^ *p* < 0.05 NALS v/s ALS; *n* = 5 in triplicates) and VEGF (***p < 0.001 NC and ^##^ *p* < 0.01 NALS v/s ALS; *n* = 5 in triplicates), respectively, upon exposure to ALS-CSF as compared to normal and disease controls. The down-regulation in mRNA expression was followed by a reduction in the protein expression for both the proteins, as observed by western blotting as well as immunofluorescence. The representative confocal images and quantitative analysis of MFI indicate a down-regulated expression of GDNF (**c**–**e**, graph **i**; **p < 0.01 NC and NALS v/s. ALS; *n* = 5 in duplicates) and VEGF (**f**–**h**, graph **j**, **p < 0.01 NC and NALS vs. ALS; *n* = 5 in duplicates), respectively, in the cultures exposed to ALS-CSF as compared to the normal controls. *Scale bars* are indicated. The dysregulation was further validated by the semi-quantitative western blot (**k**) analysis suggesting a decreased expression of both, GDNF (graph **l**; *p < 0.05 NC v/s, and ^##^ *p* < 0.01 NALS v/s. ALS; *n* = 3 in duplicates), as well as VEGF (graph **m**; *p < 0.05 NC and NALS v/s. ALS; *n* = 3 in duplicates) in response to ALS-CSF. Analysis of significance was carried out using one-way ANOVA followed by Tukey’s post hoc test

Further, to investigate the effect of ALS-CSF on the trophic factors secreted by astrocytes, we analyzed the mRNA and protein levels of GDNF and VEGF. ALS-CSF exposure resulted in near threefold down-regulation in the mRNA expression levels of GDNF in the astroglial cultures exposed to ALS-CSF for 48 h, when compared to the normal and the disease control (**p < 0.01 NC and ^#^*p* < 0.05 NALS v/s ALS; Fig. 4a). In line with the mRNA expression, the mean immunofluorescence intensity for GDNF protein was also down-regulated in the ALS group (**p < 0.01 NC and NALS v/s ALS; Fig. 4c–e, graph i). The semi-quantitative western blot analysis further validated the results (*p < 0.05 NC and ^##^*p* < 0.01 NALS v/s ALS; Fig. 4c–e, graph i).

We also observed an approximately fourfold down-regulation of VEGF mRNA level as compared to NC and NALS subsets (***p < 0.001 NC and ^##^*p* < 0.01 NALS v/s ALS; Fig. 4b). Further, the quantitative analysis of the mean fluorescence intensity also confirmed a down-regulation in the astroglial VEGF protein expression as compared to the NC and NALS subsets (**p < 0.01 NC and NALS v/s ALS, Fig. 4f–h, graph j). Similarly, a reduction in VEGF protein expression in the ALS subsets was also observed with western blot analysis (*p < 0.05 NC and NALS v/s. ALS; graph m).

### The secreted astroglial factors contributed to motor neuronal toxicity

The functional changes thus observed indicated an inflamed and toxic phenotype of astroglia, bereft of its trophic support to neurons. Therefore, to substantiate the astroglial-mediated pathology, we investigated the effect of astroglial secretions on the NSC-34 motor neuronal cell line. This was achieved by growing the NSC-34 cells in the conditioned media from the astroglial cultures exposed to either ALS-CSF (ALS-ACM) or NALS-CSF (NALS-ACM) as disease control. For the normal control, NSC-34 cells were exposed to conditioned media from the astroglial cultures grown in normal medium (NC-ACM).

**Fig. 5:**
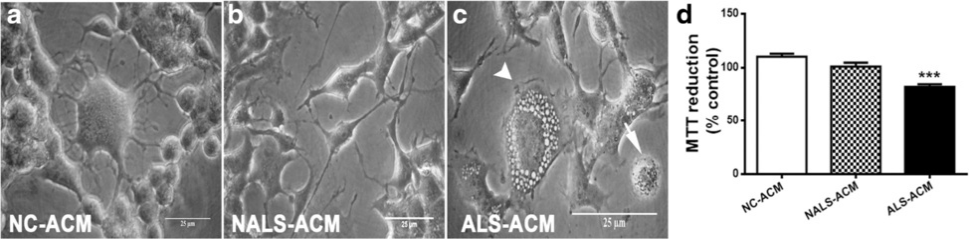
ALS-CSF conditioned astroglial medium imparts neurotoxicity to NSC-34 cells (**a**–**c**). The phase contrast images of NSC-34 cells depicting the effect of the conditioned media from astroglial cultures exposed to NALS/ALS-CSF (NALS/ALS-ACM) or NC (NC-ACM). Note the extensive vacuolation (**c**, *arrowhead*) and dying cells (**c**, *arrow*) in ALS-ACM subsets. The corresponding graph (graph **d**) shows a significant reduction in the percent cell viability as measured by altered pattern of MTT reduction, in the NSC-34 cells exposed to ALS-ACM as compared to the NC-ACM or NALS-ACM groups (***p < 0.001 NC v/s ALS; *n* = 5 in triplicates). Analysis of significance was carried out using one-way ANOVA followed by Tukey’s post hoc test. *Scale bar* is indicated

Upon exposure, ALS-ACM induced morphological changes suggestive of degeneration in NSC-34 cells, while the cells appeared healthy in NC-ACM and NALS-ACM (Fig. 5a–c). ALS-ACM induced vacuolation, clumping, and beading of neurites in NSC-34 cells. The number of undifferentiated cells increased in response to ALS-CSF exposure.

Further, there was a 25 % reduction in the cell viability in cultures exposed to ALS-ACM when compared to NALS-ACM and NC-ACM (***p < 0.001 NC-ACM and NALS-ACM v/s ALS-ACM; Fig. 5d). These results are comparable to our previous observations on the neurodegenerative potential of ALS-CSF [26].

## Discussion

Our present study outlines the inflammatory and toxic functional alterations in astrocytes in SALS and provides a composite picture of their role in exacerbating neurodegeneration. It also provides proof of the concept of an intricate pathological process with convergent pathways, involving oxidative stress, excess glutamate and nitric oxide, inflammation, and reduced trophic support thus initiating a cascade of events leading to motor neuronal insult.

Our previous studies showed that the activated astrocytes undergo hypertrophy and transform from a flat or protoplasmic morphology to a process bearing, i.e., fibrous shape, along with the down-regulation of glial glutamate transporter (GLT1) in response to ALS-CSF [39]. Here, we report that the astrocytes also undergo functional changes and contribute to inflammation-mediated toxicity. Further, qualitative observations revealed that the increase in the pro-inflammatory cytokines was more evident in the process bearing astrocytes.

Pro-inflammatory cytokines have gained notoriety for their role in triggering and propagating inflammation-mediated neurotoxicity [40]. The up-regulation of IL-6, IFN-γ, and TNF-α in both mRNA as well as protein levels in response to ALS-CSF suggests that the inflammatory factors like Chit-1, osteopontin, and other chitinases up-regulated in ALS-CSF could enhance the synthesis and secretion of these cytokines, thus promoting a vicious inflammatory cycle. Of particular relevance is the temporal sequence of cytokine release in the disease pathology. The peak expression of TNF-α and IFN-γ at 24 h and that of IL-6 at 48 h clearly establishes that IL-6 up-regulation is a downstream event. Mir and colleagues [41] noted that apart from inducing inflammatory signaling, TNF-α, and IFN-γ in the presence of inflamed glial cells are jointly capable of inducing oxidative stress and motor neuronal death. The role of IL-6 in acute phase reaction, as well as chronic inflammation, is well studied [42, 43]. Our data suggests a role for IL-6 in mediating sustained chronic inflammation in pathological conditions. Additionally, its contribution in mediating a switch from innate to adaptive immunity is well documented [44].

IL-10 is a beneficial anti-inflammatory cytokine. Vasoactive intestinal peptide (VIP) exerts its immune modulatory actions by regulating IL-10 as well as neurotrophic factors in an mSOD1 model of ALS [45]. Similarly, Ayers et al. [46] showed that IL-10 expression in the spinal axis prolonged the survival of SOD1-G93A mice. Thus, reduced expression of IL-10 protein component despite the up-regulation of its mRNA levels hints towards the inability of astrocytes to counter the ALS-CSF induced neurotoxic insult and may be denoted as a “quasi-compensatory” response, which requires further investigation. Although we did observe a trend of temporally mediated up-regulation in the IL-10 expression under normal conditions, it was relatable to the increase in basal IL-10 levels of the normal controls as reported earlier and might be a physiological, rather than experimental, phenomenon [47].

Further, pro-inflammatory cytokines can regulate the glutamatergic transmission by directly inhibiting astroglial glutamate transporters, by inducing overexpression of glutamate receptors on synapses or by reducing glutamate uptake via NO-mediated mechanism [48, 49]. Although excitotoxicity due to excess synaptic glutamate in ALS has been widely attributed to a reduction in astroglial glutamate transporters [3, 15], the possibility of enhanced glutamate secretion needed to be explored. Here, we provide objective evidence that in addition to the reduced uptake of glutamate, astroglial cells release more glutamate, which may result in its accumulation in the microenvironment of motor neurons. Reports of the microvesicular release of glutamate from astrocytes in response to insults corroborate our interpretation [50].

The inflammatory role of COX-2 and PGE-2 has been demonstrated in ALS, while their role in conferring neuroprotection or toxicity in inflammation is debated [51]. Considering the neurotoxic potential of astroglia in our study, elevated levels of COX-2 and PGE-2 could further aggravate the pathology. It is noteworthy that PGE-2, NO, and ROS are also potential effectors of glutamate release [52–54]. The excess glutamate released thus can lead to neurotoxicity through the induction of necrosis, in association with ROS, NO, and abnormal Ca^2+^ influx, as reported in the case of acute viral encephalomyelitis and PD [55].

Up-regulation of astroglial ROS, NO, and iNOS levels in response to ALS-CSF was another major finding. Elevated levels of ROS accentuate the disease pathology by causing oxidative stress, as well as reducing the glutathione (GSH) levels [56, 57]. Similarly, apart from the free radical-mediated damage, NO is capable of inflicting neurodegeneration through apoptosis, which concurs with our previous report of the occurrence of both necrosis and apoptosis, i.e., “necroptosis” induced neuronal death in the disease pathogenesis [21, 25, 26]. NO may also regulate post-translational modifications to affect the synaptic transmission and vesicular release [58, 59]. Such effects are generally brought about by excessive production of NO, mediated by either overactivation of n-NOS or pathological induction of iNOS [59]. Our results demonstrating the increased NO production and up-regulated expression of iNOS protein suggest the polarization of astrocytes to respond in a neurotoxic manner on exposure to ALS-CSF.

In the present study, we also report the capability of the conditioned medium, here representing the secreted factors, to exert neurotoxicity comparable to that of our ALS-CSF model of pathology, thus validating the role of astrocytes in the disease progression. Recently, Rojas and colleagues demonstrated that the astroglial-conditioned media from mSOD1 model brought about mitochondrial dysfunction, ROS elevation, and Ca^2+^ influx in normal glial cells, as well as neurons [60]. The ability of the conditioned media from these cultures to exert toxicity to neurons, as well as propagating pathology to glial cells, provides further proof to our own model of the propagation of pathology through the circulating fluid (ALS-CSF).

Concomitant to the inflammatory and toxic changes observed in the astrocytes, we also observed the down-regulation of the neurotrophic factors, VEGF and GDNF. They may protector reverse the motor neuron degeneration since trophic factor supplementation has been reported to be beneficial [25, 61]. Matsushita and colleagues [62] reported a significant down-regulation of GDNF in response to endotoxin LPS, suggesting its relevance in inflammation-mediated pathology. The down-regulation of these trophic factors in astrocytes, along with the previous reports of lower trophic levels in motor neurons in ALS and also during inflammation thus hint towards a possible cessation of trophic support in the inflammation-mediated neurodegeneration.

## Conclusions

In conclusion, the up-regulation of pro-inflammatory cytokines and down-regulation of anti-inflammatory cytokine suggest a clear ALS-CSF-induced astroglial cytokine imbalance. The multifarious nature of astroglial involvement in ALS pathogenesis is implied by the increase in auxiliary pro-inflammatory factors like PGE-2, COX-2, NO, and glutamate. The depletion of endogenous astrocytic VEGF and GDNF provide the additional impounding effect. Such a plethora of responses encompassing the inflammatory process suggests a temporal hierarchy of the pathways and molecules involved. Based on our results, we would like to advocate the need for combinatorial therapies to combat the multidimensional glial pathology and its compounding effect on the degeneration of motor neurons.

## Additional files

Additional file 1: Figure S1. Representative phase contrast (a, c–e) and confocal images of the enriched astroglial cultures (b, f–h). The reactive astrocytes display a process bearing morphology (e, h) as compared to the flat morphology adopted by the non-reactive astrocytes (c, f). The intermediate stages show a semi-reactive morphology with a transformation from flat to process bearing one (d, g). The cultures were found to be free of microglia as seen by the presence of GFAP immunoreactivity (red, j) and absence of Iba1 (green, i). In a similar manner, the cultures were found to be ChAT negative (green, l). The cultures were >99 % pure. Scale bars are indicated. (TIF 14438 kb) Additional file 2: Figure S2. Quantification using the inbuilt Leica software (a). The white arrowhead represents the fluorescent area selected for the intensity measurement using poly-line profile for each cell, referred to as the region of interest (ROI). Twenty cells per image were considered for each of the 10 images taken per cover slip. b The report generated by the software. The mean intensity was taken for each ROI and further analyzed. (TIF 7393 KB)

## Abbreviations

NC: Normal control
NALS: Non-amyotrophic lateral sclerosis/non-degenerative neurological diseases
ALS: Amyotrophic lateral sclerosis
IL-6: Interleukin-6
TNF: Tissue necrosis factor
IFN-γ: Interferon gamma
IL-10: Interleukin-10
NO: Nitric oxide
iNOS: Inducible nitric oxide synthase
ROS: Reactive oxygen species
PGE-2: Prostaglandin E2
COX-2: Cyclo-oxygenase 2
VEGF: Vascular endothelial growth factor
GDNF: Glial cell line-derived neurotrophic factor
NC-ACM: Normal control astroglia conditioned medium
ALS-ACM: ALS-CSF exposed astroglia conditioned medium
NALS-ACM: NALS-CSF exposed astroglia conditioned medium
NSC-34: Neuroblastoma X spinal cord hybrid cell line
ANOVA: Analysis of variance

## References

[1] Van Den Bosch L (2011). Genetic rodent models of amyotrophic lateral sclerosis. J Biomed Biotechnol.

[2] Julien JP (2007). ALS: astrocytes move in as deadly neighbors. Nat Neurosci.

[3] Rothstein JD, Van Kammen M, Levey AI, Martin LJ, Kuncl RW (1995). Selective loss of glial glutamate transporter GLT-1 in amyotrophic lateral sclerosis. Ann Neurol.

[4] Dibaj P, Steffens H, Zschuntzsch J, Kirchhoff F, Schomburg ED, Neusch C (2011). In vivo imaging reveals rapid morphological reactions of astrocytes towards focal lesions in an ALS mouse model. Neurosci Lett.

[5] Carpentier PA, Begolka WS, Olson JK, Elhofy A, Karpus WJ, Miller SD (2005). Differential activation of astrocytes by innate and adaptive immune stimuli. Glia.

[6] Jang E, Kim JH, Lee S, Kim JH, Seo JW, Jin M, Lee MG, Jang IS, Lee WH, Suk K (2013). Phenotypic polarization of activated astrocytes: the critical role of lipocalin-2 in the classical inflammatory activation of astrocytes. J Immunol.

[7] Sofroniew MV (2005). Reactive astrocytes in neural repair and protection. Neuroscientist.

[8] Zhao W, Beers DR, Appel SH (2013). Immune-mediated mechanisms in the pathoprogression of amyotrophic lateral sclerosis. J Neuroimmune Pharmacol.

[9] Pehar M, Cassina P, Vargas MR, Castellanos R, Viera L, Beckman JS, Estévez AG, Barbeito L (2004). Astrocytic production of nerve growth factor in motor neuron apoptosis: implications for amyotrophic lateral sclerosis. J Neurochem.

[10] Pekny M, Wilhelmsson U, Pekna M (2014). The dual role of astrocyte activation and reactive gliosis. Neurosci Lett.

[11] Haidet-Phillips AM, Hester ME, Miranda CJ, Meyer K, Braun L, Frakes A, Song S, Likhite S, Murtha MJ, Foust KD (2011). Astrocytes from familial and sporadic ALS patients are toxic to motor neurons. Nat Biotechnol.

[12] Tong J, Huang C, Bi F, Wu Q, Huang B, Liu X, Li F, Zhou H, Xia X-G (2013). Expression of ALS-linked TDP-43 mutant in astrocytes causes non-cell-autonomous motor neuron death in rats. EMBO J.

[13] Meyer K, Ferraiuolo L, Miranda CJ, Likhite S, McElroy S, Renusch S, Ditsworth D, Lagier-Tourenne C, Smith RA, Ravits J (2014). Direct conversion of patient fibroblasts demonstrates non-cell autonomous toxicity of astrocytes to motor neurons in familial and sporadic ALS. Proc Natl Acad Sci U S A.

[14] Lepore AC, Rauck B, Dejea C, Pardo AC, Rao MS, Rothstein JD, Maragakis NJ (2008). Focal transplantation-based astrocyte replacement is neuroprotective in a model of motor neuron disease. Nat Neurosci.

[15] Shobha K, Vijayalakshmi K, Alladi PA, Nalini A, Sathyaprabha TN, Raju TR (2007). Altered in-vitro and in-vivo expression of glial glutamate transporter-1 following exposure to cerebrospinal fluid of amyotrophic lateral sclerosis patients. J Neurol Sci.

[16] Raiteri L, Stigliani S, Zappettini S, Mercuri NB, Raiteri M, Bonanno G (2004). Excessive and precocious glutamate release in a mouse model of amyotrophic lateral sclerosis. Neuropharmacology.

[17] Milanese M, Zappettini S, Jacchetti E, Bonifacino T, Cervetto C, Usai C, Bonanno G (2010). In vitro activation of GAT1 transporters expressed in spinal cord gliosomes stimulates glutamate release that is abnormally elevated in the SOD1/G93A(+) mouse model of amyotrophic lateral sclerosis. J Neurochem.

[18] Sheng WS, Hu S, Feng A, Rock RB (2013). Reactive oxygen species from human astrocytes induced functional impairment and oxidative damage. Neurochem Res.

[19] Deepa P, Shahani N, Alladi PA, Vijayalakshmi K, Sathyaprabha TN, Nalini A, Ravi V, Raju TR (2011). Down regulation of trophic factors in neonatal rat spinal cord after administration of cerebrospinal fluid from sporadic amyotrophic lateral sclerosis patients. J Neural Transm (Vienna).

[20] Shahani N, Gourie-Devi M, Nalini A, Rammohan P, Shobha K, Harsha HN, Raju HN (2004). (−)-Deprenyl alleviates the degenerative changes induced in the neonatal rat spinal cord by CSF from amyotrophic lateral sclerosis patients. Amyotroph Lateral Scler Other Motor Neuron Disord.

[21] Shahani N, Gourie-Devi M, Nalini A, Raju TR (2001). Cyclophosphamide attenuates the degenerative changes induced by CSF from patients with amyotrophic lateral sclerosis in the neonatal rat spinal cord. J Neurol Sci.

[22] Ramamohan PY, Gourie-Devi M, Nalini A, Shobha K, Ramamohan Y, Joshi P, Raju TR (2007). Cerebrospinal fluid from amyotrophic lateral sclerosis patients causes fragmentation of the Golgi apparatus in the neonatal rat spinal cord. Amyotroph Lateral Scler.

[23] Gunasekaran R, Narayani RS, Vijayalakshmi K, Alladi PA, Shobha K, Nalini A, Sathyaprabha TN, Raju TR (2009). Exposure to cerebrospinal fluid of sporadic amyotrophic lateral sclerosis patients alters Nav1.6 and Kv1.6 channel expression in rat spinal motor neurons. Brain Res.

[24] Sharma A, Varghese AM, Vijaylakshmi K, Sumitha R, Prasanna VK, Shruthi S, Chandrasekhar Sagar BK, Datta KK, Gowda H, Nalini A (2016). Cerebrospinal fluid from sporadic amyotrophic lateral sclerosis patients induces mitochondrial and lysosomal dysfunction. Neurochem Res.

[25] Vijayalakshmi K, Ostwal P, Sumitha R, Shruthi S, Varghese AM, Mishra P, Manohari SG, Sagar BC, Sathyaprabha TN, Nalini A (2015). Role of VEGF and VEGFR2 receptor in reversal of ALS-CSF induced degeneration of NSC-34 motor neuron cell line. Mol Neurobiol.

[26] Vijayalakshmi K, Alladi PA, Sathyaprabha TN, Subramaniam JR, Nalini A, Raju TR (2009). Cerebrospinal fluid from sporadic amyotrophic lateral sclerosis patients induces degeneration of a cultured motor neuron cell line. Brain Res.

[27] Vijayalakshmi K, Alladi PA, Ghosh S, Prasanna VK, Sagar BC, Nalini A, Sathyaprabha TN, Raju TR (2011). Evidence of endoplasmic reticular stress in the spinal motor neurons exposed to CSF from sporadic amyotrophic lateral sclerosis patients. Neurobiol Dis.

[28] Rao MS, Devi MG, Nalini A, Shahani N, Raju TR (1995). Neurofilament phosphorylation is increased in ventral horn neurons of neonatal rat spinal cord exposed to cerebrospinal fluid from patients with amyotrophic lateral sclerosis. Neurodegeneration.

[29] Kulshreshtha D, Vijayalakshmi K, Alladi PA, Sathyaprabha TN, Nalini A, Raju TR (2011). Vascular endothelial growth factor attenuates neurodegenerative changes in the NSC-34 motor neuron cell line induced by cerebrospinal fluid of sporadic amyotrophic lateral sclerosis patients. Neurodegener Dis.

[30] Sankaranarayani R, Raghavan M, Nalini A, Laxmi TR, Raju TR (2014). Reach task-associated excitatory overdrive of motor cortical neurons following infusion with ALS-CSF. J Neural Transm.

[31] Sankaranarayani R, Nalini A, Rao Laxmi T, Raju TR (2010). Altered neuronal activities in the motor cortex with impaired motor performance in adult rats observed after infusion of cerebrospinal fluid from amyotrophic lateral sclerosis patients. Behav Brain Res.

[32] Varghese AM, Sharma A, Mishra P, Vijayalakshmi K, Harsha HC, Sathyaprabha TN, Bharath SM, Nalini A, Alladi PA, Raju TR (2013). Chitotriosidase—a putative biomarker for sporadic amyotrophic lateral sclerosis. Clin Proteomics.

[33] Kerstetter AE, Miller RH (2012). Isolation and culture of spinal cord astrocytes. Methods Mol Biol.

[34] Saura J (2007). Microglial cells in astroglial cultures: a cautionary note. J Neuroinflammation.

[35] Miller RG, Anderson F, Brooks BR, Mitsumoto H, Bradley WG, Ringel SP (2009). Outcomes research in amyotrophic lateral sclerosis: lessons learned from the amyotrophic lateral sclerosis clinical assessment, research, and education database. Ann Neurol.

[36] Mosmann T (1983). Rapid colorimetric assay for cellular growth and survival: application to proliferation and cytotoxicity assays. J Immunol Methods.

[37] Dong Z, Wang L, Xu J, Li Y, Zhang Y, Zhang S, Miao J (2009). Promotion of autophagy and inhibition of apoptosis by low concentrations of cadmium in vascular endothelial cells. Toxicol In Vitro.

[38] Livak KJ, Schmittgen TD (2001). Analysis of relative gene expression data using real-time quantitative PCR and the 2(−Delta Delta C(T)) Method. Methods.

[39] Shobha K, Alladi PA, Nalini A, Sathyaprabha TN, Raju TR (2010). Exposure to CSF from sporadic amyotrophic lateral sclerosis patients induces morphological transformation of astroglia and enhances GFAP and S100beta expression. Neurosci Lett.

[40] Hooten KG, Beers DR, Zhao W, Appel SH (2015). Protective and toxic neuroinflammation in amyotrophic lateral sclerosis. Neurotherapeutics.

[41] Mir M, Asensio VJ, Tolosa L, Gou-Fabregas M, Soler RM, Lladó J, Olmos G (2009). Tumor necrosis factor alpha and interferon gamma cooperatively induce oxidative stress and motoneuron death in rat spinal cord embryonic explants. Neuroscience.

[42] McLoughlin RM, Jenkins BJ, Grail D, Williams AS, Fielding CA, Parker CR, Ernst M, Topley N, Jones SA (2005). IL-6 trans-signaling via STAT3 directs T cell infiltration in acute inflammation. Proc Natl Acad Sci U S A.

[43] Rath T, Billmeier U, Waldner MJ, Atreya R, Neurath MF (2015). From physiology to disease and targeted therapy: interleukin-6 in inflammation and inflammation-associated carcinogenesis. Arch Toxicol.

[44] Kaplanski G, Marin V, Montero-Julian F, Mantovani A, Farnarier C (2003). IL-6: a regulator of the transition from neutrophil to monocyte recruitment during inflammation. Trends Immunol.

[45] Goursaud S, Schafer S, Dumont AO, Vergouts M, Gallo A, Desmet N, Deumens R, Hermans E (2015). The anti-inflammatory peptide stearyl-norleucine-VIP delays disease onset and extends survival in a rat model of inherited amyotrophic lateral sclerosis. Exp Neurol.

[46] Ayers JI, Fromholt S, Sinyavskaya O, Siemienski Z, Rosario AM, Li A, Crosby KW, Cruz PE, DiNunno NM, Janus C (2015). Widespread and efficient transduction of spinal cord and brain following neonatal AAV injection and potential disease modifying effect in ALS mice. Mol Ther.

[47] Gonzalez P, Burgaya F, Acarin L, Peluffo H, Castellano B, Gonzalez B (2009). Interleukin-10 and interleukin-10 receptor-I are upregulated in glial cells after an excitotoxic injury to the postnatal rat brain. J Neuropathol Exp Neurol.

[48] Pickering M, Cumiskey D, O’Connor JJ (2005). Actions of TNF-alpha on glutamatergic synaptic transmission in the central nervous system. Exp Physiol.

[49] Hu S, Sheng WS, Ehrlich LC, Peterson PK, Chao CC (2000). Cytokine effects on glutamate uptake by human astrocytes. Neuroimmunomodulation.

[50] Bergersen LH, Gundersen V (2009). Morphological evidence for vesicular glutamate release from astrocytes. Neuroscience.

[51] Consilvio C, Vincent AM, Feldman EL (2004). Neuroinflammation, COX-2, and ALS—a dual role?. Exp Neurol.

[52] Bal-Price A, Brown GC (2001). Inflammatory neurodegeneration mediated by nitric oxide from activated glia-inhibiting neuronal respiration, causing glutamate release and excitotoxicity. J Neurosci.

[53] Bezzi P, Carmignoto G, Pasti L, Vesce S, Rossi D, Rizzini BL, Pozzan T, Volterra A (1998). Prostaglandins stimulate calcium-dependent glutamate release in astrocytes. Nature.

[54] Socodato R, Portugal CC, Canedo T, Domith I, Oliveira NA, Paes-de-Carvalho R, Relvas JB, Cossenza M (2015). c-Src deactivation by the polyphenol 3-O-caffeoylquinic acid abrogates reactive oxygen species-mediated glutamate release from microglia and neuronal excitotoxicity. Free Radicg Biol Med.

[55] Amor S, Puentes F, Baker D, van der Valk P (2010). Inflammation in neurodegenerative diseases. Immunology.

[56] Weiduschat N, Mao X, Hupf J, Armstrong N, Kang G, Lange DJ, Mitsumoto H, Shungu DC (2014). Motor cortex glutathione deficit in ALS measured in vivo with the J-editing technique. Neurosci Lett.

[57] Vargas MR, Johnson DA, Johnson JA (2011). Decreased glutathione accelerates neurological deficit and mitochondrial pathology in familial ALS-linked hSOD1(G93A) mice model. Neurobiol Dis.

[58] Bavencoffe A, Chen SR, Pan HL (2014). Regulation of nociceptive transduction and transmission by nitric oxide. Vitam Horm.

[59] Bradley SA, Steinert JR (2016). Nitric oxide-mediated posttranslational modifications: impacts at the synapse. Oxid Med Cell Longev.

[60] Rojas F, Gonzalez D, Cortes N, Ampuero E, Hernández DE, Fritz E, Abarzua S, Martinez A, Elorza AA, Alvarez A (2015). Reactive oxygen species trigger motoneuron death in non-cell-autonomous models of ALS through activation of c-Abl signaling. Front Cell Neurosci.

[61] Henderson CE, Phillips HS, Pollock RA, Davies AM, Lemeulle C, Armanini M, Simmons L, Moffet B, Vandlen RA, Simpson LCctSL (1994). GDNF: a potent survival factor for motoneurons present in peripheral nerve and muscle. Science.

[62] Matsushita Y, Nakajima K, Tohyama Y, Kurihara T, Kohsaka S (2008). Activation of microglia by endotoxin suppresses the secretion of glial cell line-derived neurotrophic factor (GDNF) through the action of protein kinase C alpha (PKCalpha) and mitogen-activated protein kinases (MAPKS). J Neurosci Res.

