# Supplementary figures and images for: Astroglia acquires a toxic neuroinflammatory role in response to the cerebrospinal fluid from amyotrophic lateral sclerosis patients
Source: J Neuroinflammation. 2016 Aug 30;13(1):212. doi: 10.1186/s12974-016-0698-0 (PMC5006495; doi:10.1186/s12974-016-0698-0)

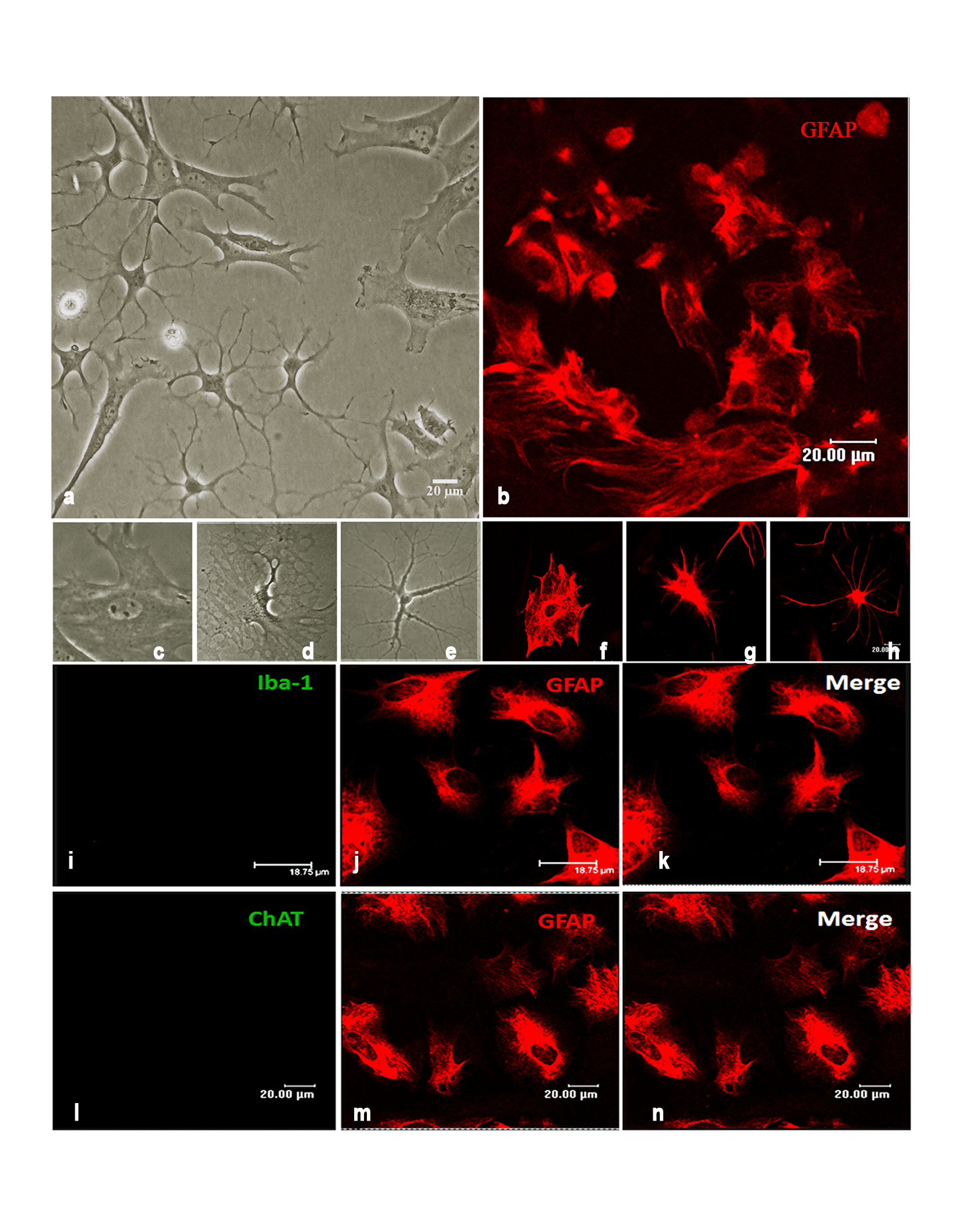

Supplement: Additional file 1: Figure S1. — Representative phase contrast (a, c–e) and confocal images of the enriched astroglial cultures (b, f–h). The reactive astrocytes display a process bearing morphology (e, h) as compared to the flat morphology adopted by the non-reactive astrocytes (c, f). The intermediate stages show a semi-reactive morphology with a transformation from flat to process bearing one (d, g). The cultures were found to be free of microglia as seen by the presence of GFAP immunoreactivity (red, j) and absence of Iba1 (green, i). In a similar manner, the cultures were found to be ChAT negative (green, l). The cultures were >99 % pure. Scale bars are indicated. (TIF 14438 kb) [file 12974_2016_698_MOESM1_ESM.tif]

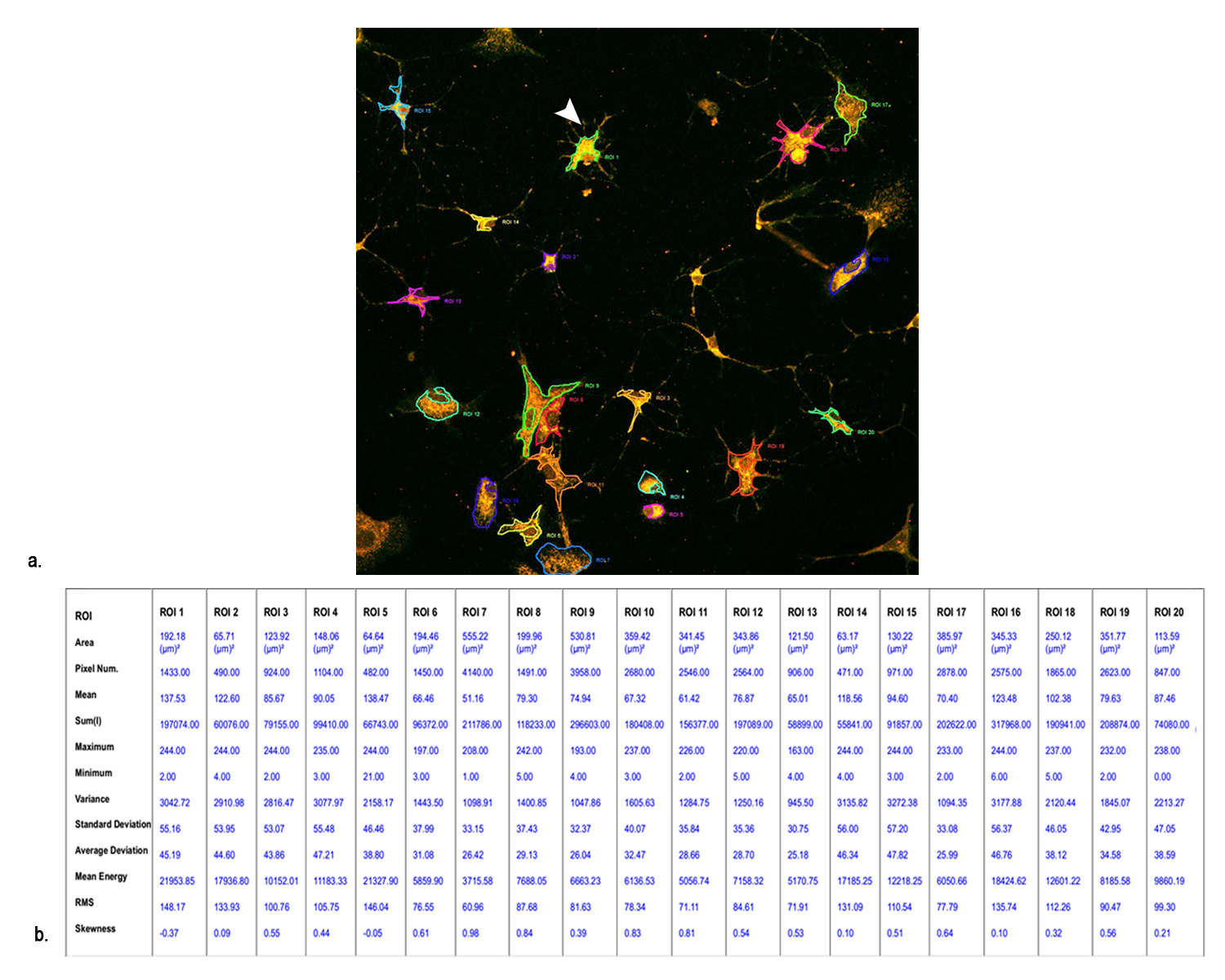

Supplement: Additional file 2: Figure S2. — Quantification using the inbuilt Leica software (a). The white arrowhead represents the fluorescent area selected for the intensity measurement using poly-line profile for each cell, referred to as the region of interest (ROI). Twenty cells per image were considered for each of the 10 images taken per cover slip. b The report generated by the software. The mean intensity was taken for each ROI and further analyzed. (TIF 7393 KB) [file 12974_2016_698_MOESM2_ESM.tif]
